# Response rate and costs for automated patient-reported outcomes collection alone compared to combined automated and manual collection

**DOI:** 10.1186/s41687-019-0121-6

**Published:** 2019-06-03

**Authors:** Yvette Pronk, Peter Pilot, Justus M. Brinkman, Ronald J. van Heerwaarden, Walter van der Weegen

**Affiliations:** 1 Research Department, Kliniek ViaSana, Mill, The Netherlands; 2 Zimmer Biomet NL, Dordrecht, The Netherlands; 3 Department of orthopaedic surgery, Kliniek ViaSana, Mill, The Netherlands; 4 Department of orthopaedic surgery, Sint Anna Ziekenhuis, Geldrop, The Netherlands

**Keywords:** Patient-reported outcome measurements, Response rate, Costs

## Abstract

**Background:**

The response rate on patient-reported outcome measurements (PROMs) necessary to adequately evaluate a treatment and improve patient care is unknown. Hospitals generally aim for the highest possible response rate without insight into the increase in costs involved. This study aimed to investigate which PROMs response rate is achievable in relation to the costs in an orthopaedic practice.

**Methods:**

In an observational study, patients planned for orthopaedic surgery were asked to participate per surgical procedure (5769 surgical procedures at 5300 patients). Patient-reported outcomes (PROs) collection with a digital online automated PROMs collection system (minimal effort) was compared to a combined automated system and manual collection (maximal effort). Response rate was calculated preoperative and at two postoperative time points separately, and on all three time points together. Costs were calculated for the study period, per year and per surgical procedure. Calculations were executed for all surgical procedures and for three subgroups: knee arthroplasty, hip arthroplasty and anterior cruciate ligament reconstruction (ACLR).

**Results:**

Using maximal effort the response rate increased for all surgical procedures compared to minimal effort; the preoperative response rate from 86% to 100% and the postoperative response rates from 55% to 83% (3 or 6 months) and 53% to 83% (12 months). Concerning the response at all three time points for all surgical procedures, minimal effort resulted in 44% response rate and increased to 76% with maximal effort. Lowest postoperative response rates were found in the ACLR group for both maximal and minimal effort. A costs difference of €5.55–€5.98 per surgical procedure between maximal and minimal effort was found.

**Conclusions:**

A two times higher PROMs response rate for patients responding at all three time points (44% versus 76%) is achievable with maximal effort compared to the use of an automated PROMs collection system only. Manual collection adds a cost of €5.5–€6 per surgical procedure to automated PROMs collection alone. It is debatable if these additional costs are justifiable from a value-based health care perspective as the response rate for adequate evaluation of a treatment is still unknown.

## Background

From a patient’s perspective, implant survival may not be the best measure of surgery success. Instead, pain reduction, functional improvement and quality of life are important [1–4]. With this shift towards a more patient-centered perspective in health care, there is an increase in the use of Patient-Reported Outcome Measurements (PROMs) [5]. PROMs are questionnaires that assess health status from patient’s perspective and focus on pain, function, quality of life and/or satisfaction. This has resulted in the addition of patient-reported outcomes (PROs) to (national) arthroplasty registries for evaluating treatments and improving patient care. Since 2007 all Dutch hospitals have registered their implanted prostheses in a national registry and in 2012 the Dutch Orthopaedic Association (NOV) advised hospitals to add PROs collected by selected PROMs [6, 7]. This resulted in the first PROMs indicator which obliges hospitals to collect PROs of all hip arthroplasty patients. The first part of this indicator is a process indicator as it focusses on the achieved response rate.

To achieve the goal of evaluating treatments and improving patient care a certain level of response rate is necessary to ensure generalizability and to minimize selection bias of the collected PROs [8]. Unfortunately, there is no clear consensus of what rate is acceptable. The International Society of Arthroplasty Registries (ISAR) PROMs Working Group proposed a response rate of at least 60% [9, 10]. That percentage is based on what is considered a sufficient response rate in survey research [11]. In 2017, the Dutch arthroplasty registry reported an average preoperative response rate of 54%, ranging from 5% to 99% [12].

Although PROs are an important component of health outcome and several authors have reported tips and tricks regarding PROs collection [13–15], even specific for orthopaedic practice [9, 16], this wide range in response rate reported by the Dutch arthroplasty registry shows that the implementation and integration of PROs collection into orthopaedic practice has its challenges. Generally, hospitals strive for an as high as possible response rate without having an insight into the increase in costs involved and not knowing if their response rate justifies the expenses made.

Therefore, a clear understanding is needed of which response rate is achievable and at what costs. The aim of this study was to investigate which PROMs response rate is achievable in relation to the costs for PROs collection in an orthopaedic practice.

## Methods

### Setting and inclusion

PROs collection was performed in a medium–sized-orthopaedic hospital (Kliniek ViaSana, Mill, the Netherlands). Between January 2014 and June 2015, 5300 orthopaedic patients that underwent in total 5769 surgical procedures, characterised by aged 12 years and older, American Society of Anaesthesiologists (ASA) classification of I or II, and body mass index (BMI) ≤35 kg/m^2^, were followed.

Patients were informed and asked by their surgeon’s receptionist to participate in PROs collection and to allow further scientific analysis using their anonymised data. All included patients signed the informed consent form. PROMs sets were based on the type of surgery performed and included those that were mandatory as set out by the NOV [10]. All sets had comparable length and linguistic difficulty. Retrospective analysis was executed on the prospectively collected data. This study was approved by the district medical ethics committee (N18.156).

### Data collection

Patients registered and completed their preoperative PROMs on a computer using a web-based survey of a digital, online, automated system for collecting PROs (OnlinePROMs, Interactive Studios, Rosmalen, the Netherlands) directly after consultation in the hospital. In case they needed assistance or could not handle a computer, an (admission administrator) employee was available to provide instructions or hand out paper forms. Before surgery, completeness of the PROMs was checked by the PROMs administrator and in case of incomplete PROMs, a paper form was handed out to the patient at the day of surgery to collect the missing PROs (response check). After surgery the PROMs administrator manually entered the date of surgery in the automated system; by doing so, postoperative PROMs were automatically sent by email 3 or 6, and 12 months after surgery. In case of non-response an automatic reminder was sent after 7 days. In case no email address was registered, the PROMs were sent by postal service and included an invitation letter and a stamped self-addressed envelope. This was all done by the PROMs administrator who received a notification by the automated system to execute this. If the patient did not respond after two invitations by email, the PROMs administrator automatically received a notification by the system to send a third invitation per postal service. All returned forms were manually entered in the automated system by the PROMs administrator. All questions in the automated system were mandatory. In total, per surgical procedure the patient was invited to complete the PROMs at three time points: preoperatively, at 3 or 6 months postoperatively, and at 12 months postoperatively.

### Data analysis

After data collection, per surgical procedure and per time point patients were allocated to two groups: the minimal effort or the maximal effort group. Patients for which PROs were collected only using the automated system were included in the minimal effort group. For this group, additional manual labour was only needed for entering the date of surgery. The maximal effort group included all patients where extra manual labour was needed: response check, PROMs sent by postal service, third invitations sent by postal service and remaining tasks. These remaining tasks consisted of answering patients phone calls or emails, or correcting administrative errors such as wrong email addresses.

#### Response rate and costs

Response rate was calculated by dividing the number of returned questionnaires completed partly or totally by the number of surgical procedures minus the number of surgical procedures of patients who were deceased (returned questionnaires / (surgical procedures – surgical procedures of patients who were deceased)) [9]. Reasons for loss to follow-up were reported. First, response rate was calculated per time point. Second, it was calculated for all three time points together. Response at all three time points was defined as when per surgical procedure a patient returned the PROMs at all three time points: preoperatively, 3 or 6 months postoperatively, ánd 12 months postoperatively. When there was no returned questionnaire on one or more time points, this was defined as no response at all three time points. Completion rate per time point was calculated by dividing the number of returned questionnaires completed totally by the number of surgical procedures minus the number of surgical procedures of patients who were deceased (totally completed returned questionnaires / (surgical procedures – surgical procedures of patients who were deceased)). Costs were calculated for the entire study period, per surgical procedure and per year. Costs consisted of the license fee for the automated PROMs system (€7500,- per year), pay for two computers on which the registration and completion of the preoperative PROMs was done (€1600,- over 5 years), costs for paper forms including sending per postal service (€0.08 per sheet of paper, €0.07 per envelope and €10.000 per year for sending), and staff employment costs: PROMs administrator (€22.1 per hour), surgeon’s receptionist (€21.1 per hour) and admission administrator (€22.1 per hour). The amount of time needed for all specific manual tasks in the collection process was estimated. Response rate and costs were calculated for all surgical procedures and for three patient groups as subgroups: total hip arthroplasty (THA), total or unicompartmental knee arthroplasty (TKA&UKA) and anterior cruciate ligament reconstruction (ACLR). Baseline demographic data were collected from the electronic patient records.

## Results

**Fig. 1:**
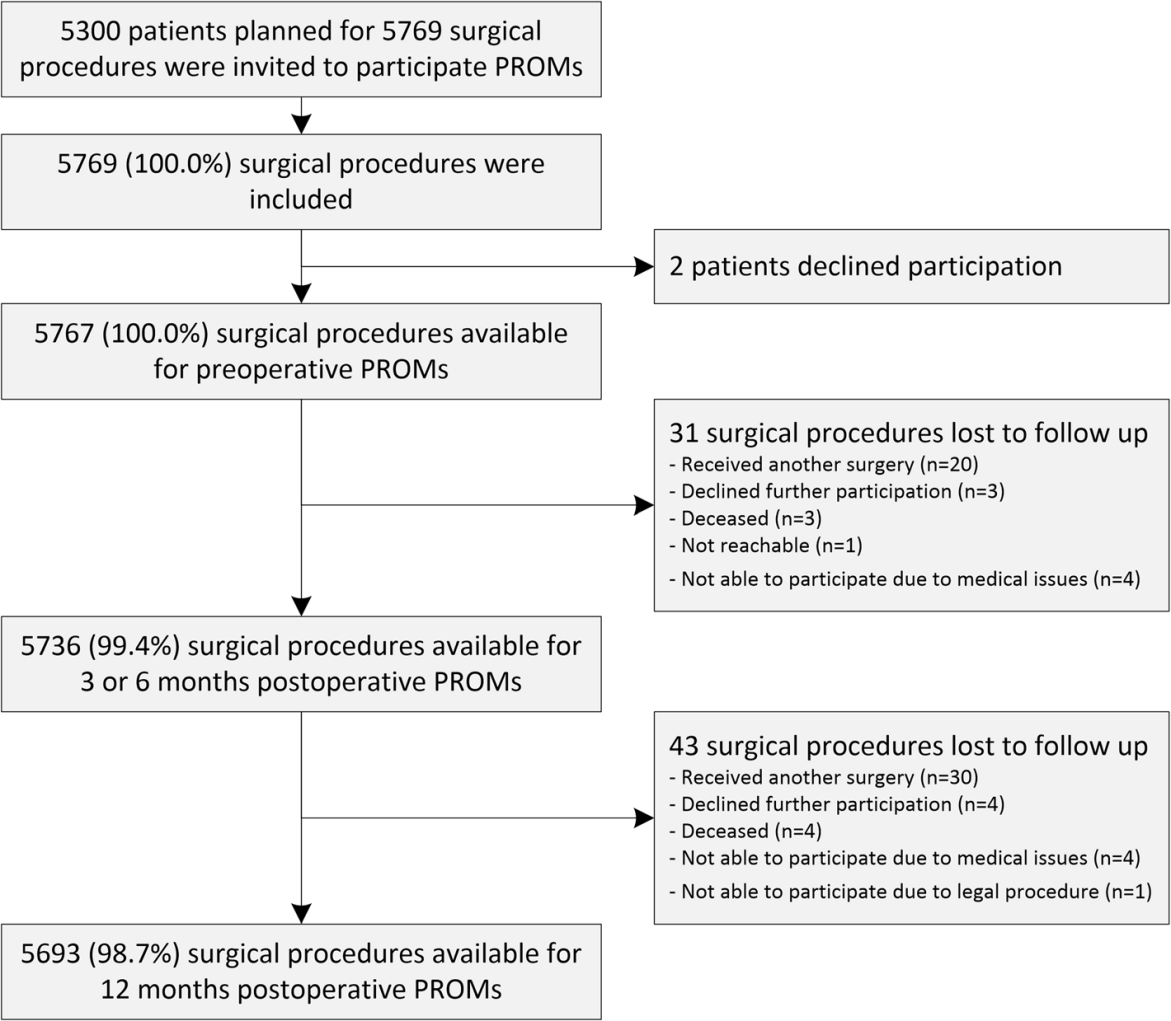
Flowchart . *Note*: *PROMs* indicates patient-reported outcome measurements. *n* indicates number

Between January 2014 and June 2015, all 5300 patients planned for 5769 surgeries were included of which only 2 times a patient declined participation, therefore 5767 surgical procedures (100%) of 5298 patients were available for participating PROMs (Fig. 1).

### Characteristics

The characteristics of the 5769 surgical procedures as well as the subgroups qualifications are listed in Table 1.

**Table 1:** Characteristics for all surgical procedures and the THA, TKA&UKA and ACLR subgroups

	All surgical procedures (*n* = 5769)	THA (*n* = 535)	TKA&UKA (*n* = 742)	ACLR (*n* = 430)
Age (y, mean ± SD)	50.3 ± 15.8	64.7 ± 8.3	64.3 ± 7.8	27.4 ± 9.5
BMI (kg/m^2^, mean ± SD)	26.0 ± 3.6	25.9 ± 3.5	28.0 ± 3.5	23.8 ± 2.9
Gender – female (n, (%))	2715 (47.1%)	339 (63.4%)	377 (50.8%)	138 (32.1%)
ASA – II (n, (%))	1986 (34.4%)	264 (49.4%)	438 (59.0%)	25 (5.81%)

*Note*: *THA* indicates total hip arthroplasty, *TKA* indicates total knee arthroplasty, *UKA* indicates unicompartimental knee arthroplasty, *ACLR* indicates anterior cruciate ligament reconstruction, *y* indicates year, *SD* indicates standard deviation, *BMI* indicates body mass index, *kg/m*^*2*^ indicates kilogram per square meter, *n* indicates number, *ASA* indicates American Society of Anaesthesiologists classification

### Response rate

**Fig. 2:**
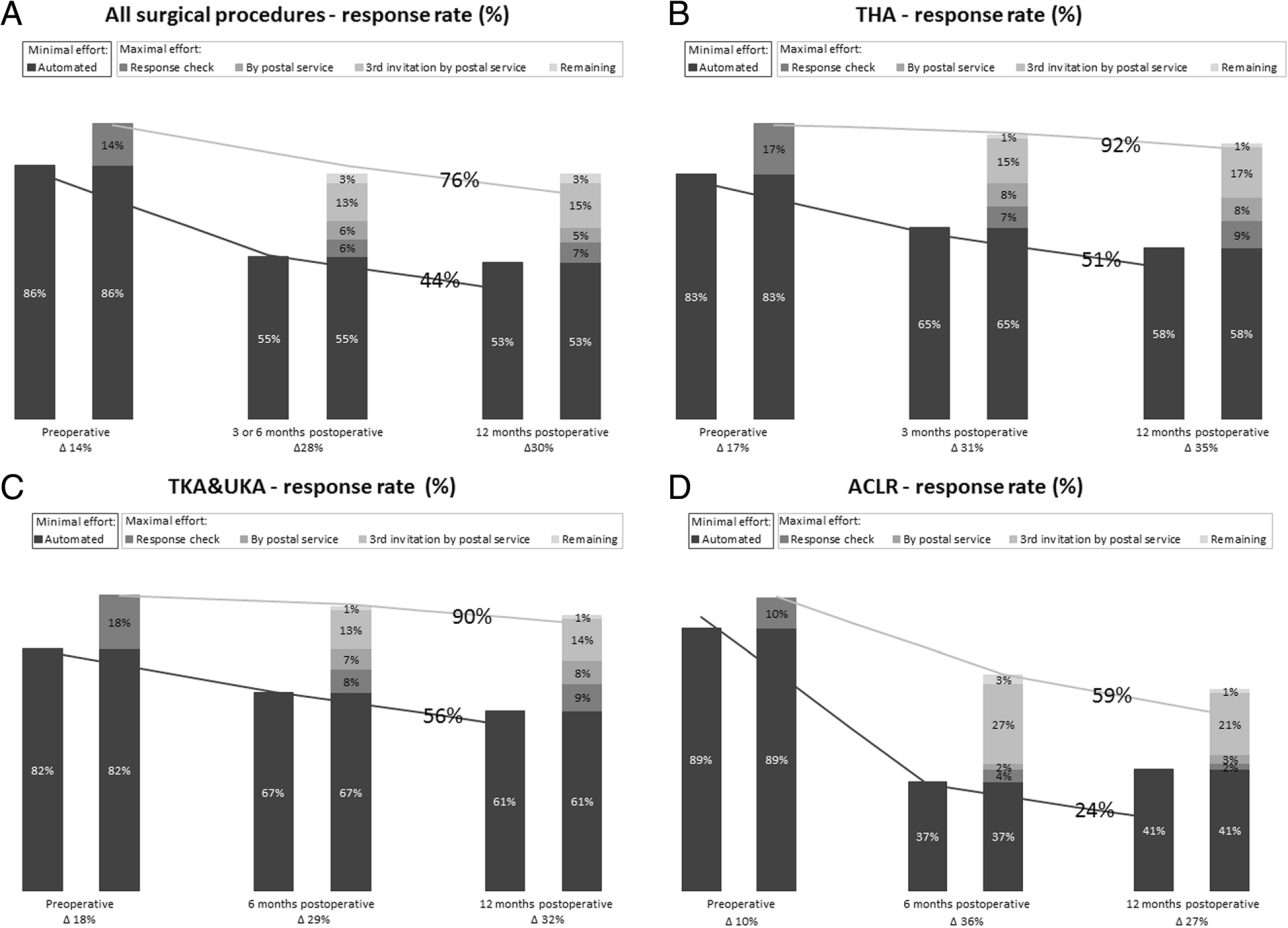
Response rates (%) of PROs collection: minimal effort versus maximal effort. Represented for all surgical procedures (**a**) and the THA (**b**), TKA&UKA (**c**) and ACLR (**d**) subgroups. The line represents the response rate at all three time points. *Note*: *PROs* indicates patient-reported outcomes. *THA* indicates total hip arthroplasty. *TKA* indicates total knee arthroplasty. *UKA* indicates unicompartimental knee arthroplasty. *ACLR* indicates anterior cruciate ligament reconstruction

With maximal effort for PROs collection the response rate increased for all surgical procedures compared to minimal effort, the preoperative response rate from 86% to 100% and the postoperative response rates from 55% to 83% (3 or 6 months) and 53% to 83% (12 months) (Fig. 2a). The lowest postoperative response rates were found in the ACLR group for both maximal and minimal effort compared to the other groups (Fig. 2). For all surgical procedures minimal effort resulted in 44% response rate at all three time points. An increased in response rate to 76% was reached with maximal effort (Fig. 2a). Various differences in response rates between the subgroups were found (Fig. 2b-d).

Of all the additional tasks performed in the maximal effort group sending a third invitation by postal service after no response on two automated email invitations resulted in the highest extra response rate in all surgical procedures and in all the three subgroups ranging from 13% to 27% (Fig. 2).

Regarding the completion rate, maximal effort for PROs collection resulted in 100% preoperative completion rate compared to 86% with minimal effort, 81% compared to 54% 3 or 6 months postoperatively and 79% in comparison with 52% 12 months postoperatively respectively.

### Costs

Costs associated with collecting PROs with maximal effort for all surgical procedures increased to €56,081 compared to €23,079 with minimal effort; €9.72 versus €4.00 per surgical procedure and €37,481 versus €15,479 per year. In all surgical procedures and in the three subgroups, the calculated difference per surgical procedure between minimal and maximal effort ranged between €5.55 and €5.98. Costs per surgical procedure in the three subgroups were the highest in the ACLR group for both minimal (€28.44) and maximal effort (€34.42) compared to the other subgroups (Table 2).

**Table 2:** Costs of PROs collection: minimal effort versus maximal effort

	All surgical procedures (*n* = 5769)		THA (*n* = 535)		TKA&UKA (*n* = 742)		ACLR (*n* = 430)	
	Minimal effort	Maximal effort	Minimal effort	Maximal effort	Minimal effort	Maximal effort	Minimal effort	Maximal effort
License fee for automated system (€)	10,875	10,875	10,875	10,875	10,875	10,875	10,875	10,875
Two computers for registration and completion of the preoperative PROMs (€)	480	480	480	480	480	480	480	480
Staff (€)								
PROMs administrator	1725	17,254	160	1599	217	2165	129	1285
Surgeon’s receptionist	2027	2027	188	188	261	261	151	151
Admission administrator	7510	7510	696	696	966	966	560	560
Paper forms and sending (€)	462	17,936	43	1573	59	2264	34	1447
In total (€)	23,079	56,081	12,442	15,411	12,857	17,011	12,229	14,799
Per surgical procedure (€)	4.00	9.72	23.26	28.81	17.33	22.93	28.44	34.42
Per year (€)	15,479	37,481	8283	10,263	8581	11,350	8176	9889

Represented for all surgical procedures and the THA, TKA&UKA and ACLR subgroups

*Note: PROs* indicates patient-reported outcomes, *THA* indicates total hip arthroplasty, *TKA* indicates total knee arthroplasty, *UKA* indicates unicompartimental knee arthroplasty, *ACLR* indicates anterior cruciate ligament reconstruction, *€* indicates euro, *n* indicates number

## Discussion

This study aimed to investigate which PROMs response rate is achievable in relation to the costs for PROs collection in an orthopaedic practice. Collecting PROs with maximal effort for all surgical procedures resulted in a preoperative response rate increasing from 86% reachable with minimal effort to the optimal of 100%, and at the two postoperative time points from 53% or 55% to 85%. Furthermore, with maximal effort a two times higher response rate for patients responding at all three time points was achievable compared to only using a digital online automated PROMs collection system as minimal effort. Both achieved with two times higher costs (€4 to €10 per surgical procedure). These additional costs of €6 per surgical procedure were found for all surgical procedures as well as in the subgroups. Regarding these subgroups, lowest response rates and highest costs were found in the ACLR group with both maximal and minimal effort.

The only two previous orthopaedic studies that use a digital online automated PROMs collection system reported 43% response 6 months after knee surgery for patellar instability, ligament, cartilage, or meniscus injury [17] and 92% after elbow arthroplasty [18]. Howard et al. found similar rates related to the ACLR patients (37%) as the most comparable group of the current study. However, only 9% of their patients responded at all time points [17], which is less compared to the 24% in the present study. Viveen et al. used the same automated system and reported a similar response rate to this study, but calculated it by dividing the number of returned PROMs by the number of sent PROMs [18]. In studies outside of orthopaedics, response rates of web-based surveys vary greatly between 14% and 83% [19–23]. Web-based surveys are said to be cost-effective [14], have a decreased risk of errors and missing values [24] and are favoured [25] compared to paper forms. In the current study, only using an automated system, the ISAR PROMs Working Group proposed response rate of at least 60% was reached for the preoperative collected PROs [9], but not postoperatively for all surgical procedures, ACLR and THA at 12 months. Regarding at least 60% on all preoperative ánd postoperative time points, none of the four groups reached this threshold while using an automated system only. Using maximal effort in collecting PROs this ISAR threshold is almost achieved as it resulted in at least 68% for one single time point and at least 59% response at all three time points. This shows that alternatives beside an automated system as minimal effort to complete PROMs are needed to improve response rate [14, 26, 27] and to reach the proposed threshold of 60%. Similarly, Rolfson et al. concluded that only using web-based surveys in THA patients results in an insufficient response rate of 49%, and it is unable to replace PROs collection with paper forms in PROs collection with an automated system only as the PROs and patient demographics for being a respondent differ between both ways of collection [24]. The sending of a 3rd invitation by postal service after no response was received on two email invitations, as a part of maximal effort, had the highest impact (≥13% extra response rate) on improving postoperative response rate and should be added to any automated collection system in order to achieve the ISAR threshold on every single time point. To achieve the proposed threshold for response at all three time points, maximal effort is needed. The downside of this is that maximal effort increased costs.

A recent study among trauma and orthopaedic surgeons concluded that one of the two most important constraints against implementing PROMs was costs [28]. Previous studies reported $2.00–$6.39 (€1.70–€5.50) per respondent using an automated system [19, 29] reaching a lower response rate (between 14% and 21%) compared to the current study. In the present study, collecting PROs was €6 per surgical procedure more expensive with maximal effort. The smaller the number of surgical procedures, the fixed costs such as the license fee for an automated system and hardware weigh heavier, as shown by the smaller ACLR group that was more expensive per surgical procedure compared to all surgical procedures included. Therefore, to consider the value of adding costs of €6 per surgical procedure to achieve higher response rate, the size of the hospital or patient group involved should be taking into account. Regarding the different patient groups, the THA and TKA&UKA patients had the highest pre- and postoperative response rates and had the lowest costs to collect PROs. This might be explained by their more compliant attitude to their surgeon [30]. The younger ACLR patients showed to be more inclined to handle computers due to their high preoperative response rate by using only an automated system [19]. However, their postoperative response rates with an automated system only were lower compared to the older patient groups. It might be that the age group of ACLR patients already get too many emails, so they were more aware of responding due to an invitation by postal service, as seen in the higher response rates on a 3rd invitation by postal service. Furthermore, the ACLR patients were mainly male patients who are reported to be more likely to respond by postal service [19, 26]. Younger [18, 19, 31] and male [19, 30, 31] patients in general are the most challenging group; they are less likely to respond at all. This also explains the higher costs for the ACLR patient in the current study. To ensure wider acceptance and to improve the response rate, postal service as additional effort is advised in younger and male patients [14, 26, 27]; again with the downside of higher costs.

Little is known about the costs made to collect PROs in relation to the benefit of collecting PROs. The present study shows the considerable costs to achieve high response rates; knowing that these costs are even without costs for data analysis and improvement strategies, which is expected to result in reducing costs. From a value based health care perspective, it is questionable if the costs made to collect PROs, and the additional costs for improving the response rate, are justifiable. The most important question might not be how many response is needed, but how representative the respondents are for the hospital or patient group in question [32]. It could very well be that a more homogeneous patient population in a specific setting requires a lower response rate compared to a more heterogeneous patient population in another setting. It is questionable that a quality indicator is set on achieved response rate without actually knowing the threshold.

To the authors knowledge, this is the first study clarifying the achievable response rate on PROMs versus the associated health care costs in a medium sized orthopaedic practice. It provides other hospitals insights into what costs they might expect for collecting PROs in their hospital setting or patient groups using minimal and maximal effort. A limitation of this study was that the amount of time needed for all specific manual tasks in the collection process was not exactly measured but was estimated.

## Conclusions

A two times higher PROMs response rate for patients responding at all time points is achievable with maximal effort compared to the use of a digital online automated PROMs collection system only for PROs collection in an orthopaedic practice. Manual collection adds a cost of €6 per surgical procedure to automated PROMs collection alone. As the response rate for adequate evaluation of a treatment is still unknown it is questionable if these additional costs are justifiable from a value-based health care perspective.

## Abbreviations

ACLR: Anterior Cruciate Ligament Reconstruction
ASA: American Society of Anaesthesiologists
BMI: Body Mass Index
ISAR: International Society of Arthroplasty Registries
NOV: Dutch Orthopaedic Association
PROMs: Patient-Reported Outcome Measurements
PROs: Patient-Reported Outcomes
THA: Total Hip Arthroplasty
TKA: Total Knee Arthroplasty
UKA: Unicompartimental Knee Arthroplasty
